# Boundary conditions for exploiting the cooperation of *Aminobacter niigataensis* MSH1 with *Piscinibacter* sp. K169 to support 2,6-dichlorobenzamide biodegradation in sand filters for drinking water treatment: role of cell density and organic carbon

**DOI:** 10.1128/aem.01149-25

**Published:** 2025-09-25

**Authors:** Siyao Du, Aura Wouters, Manon Glorieux, Laurien van Lieshout, Benjamin Horemans, Dirk Springael

**Affiliations:** 1 Division of Soil and Water Management, KU Leuven 26657, Heverlee, Belgium; University of Milano-Bicocca, Milan, Italy

**Keywords:** drinking water treatment, bioaugmentation, micropollutant biodegradation, bacterial cooperation, sand filter microcosm

## Abstract

*Aminobacter niigataensis* MSH1 mineralizes the groundwater micropollutant 2,6-dichlorobenzamide (BAM) and is a candidate for bioaugmentation of sand filters in drinking water treatment plants (DWTP) to avert BAM-contamination. *Piscinibacter* sp. K169 is a sand filter isolate that improves MSH1-mediated BAM mineralization through a cooperative interaction, and co-inoculation of MSH1 with K169 is proposed as a strategy to support bioaugmentation with MSH1. In this study, boundaries regarding the initial population size and the supply of organic carbon resources determining the interaction between MSH1 and K169 in sand filter microcosms were explored. The cooperative interaction was only disturbed when initial cell densities of one of the two partners were 10^4^ cells/mL or lower. Supplying acetate as a carbon source appeared redundant for supporting BAM mineralization. Instead, the organic carbon present on the sand drove the cooperative interaction between K169 and MSH1 as the effect of K169 on BAM mineralization disappeared, and none of the two strains showed growth in sand devoid from organic carbon. These findings highlight the feasibility of K169-assisted bioaugmentation with MSH1 under realistic field conditions, as it requires no supplementary organic carbon and remains effective, even at relatively low inoculum densities, thereby addressing key challenges in bioaugmentation strategies.

**IMPORTANCE:**

Bioaugmentation of sand filters exploited in drinking water treatment, with the BAM catabolic strain *Aminobacter niigataensis* MSH1, has previously been successful during the first 1–2 weeks, where after BAM degradation deteriorated together with the loss of MSH1 cell density and cell activity. Bacterial isolates obtained from sand filters can support BAM degradation activity by MSH1 involving mutualistic interactions which resulted in the proposition of a novel bioaugmentation approach involving the co-inoculation of “support” bacteria that are adapted to the target environment. This paper focuses on understanding the boundary conditions required for sustaining the mutualistic interaction between MSH1 and such a “supportive” sand filter isolate in sand microcosm, showing that the interaction could be maintained when using relatively low cell densities and with no additional carbon supplemented. To the best of our knowledge, this paper is the first study to examine the boundary conditions of a bacterial mutualistic interaction, particularly in a bioaugmentation context of water treatment.

## INTRODUCTION

2,6-Dichlorobenzamide (BAM), a transformation product of the herbicide dichlobenil (2,6-dichlorobenzonitrile), is a frequently detected groundwater micropollutant that poses a challenge for drinking water treatment plants (DWTPs) (1). *Aminobacter niigataensis* MSH1 is a bacterial soil isolate that aerobically mineralizes BAM at micro-concentrations. The organism has been extensively characterized for its BAM catabolism at the genetic and biochemical levels and was proposed for bioaugmentation of filtration units exploited in DWTPs, such as rapid sand filters, to avert BAM contamination (2–5). Bioaugmentation with MSH1 resulted in the successful removal of BAM in groundwater from 0.2 µg/L to below the legally mandated 0.1 µg/L threshold for drinking water (Council Directives 98/83/EC and 2006/118/EC) in laboratory and pilot sand filters (6, 7). However, BAM biodegradation deteriorated over time due to the decrease in MSH1 cell density and activity likely linked to limitations in carbon and energy resources inherent to the oligotrophic target environment (6–8).

Co-inoculation of MSH1 with organisms that (i) originate from the target environment (i.e., oligotrophic DWTP sand filter) and (ii) support the degraders’ activity, was recently proposed as an approach to improve bioaugmentation (9). Vandermaesen et al. (9) identified DWTP sand filter resident bacteria, such as *Piscinibacter* sp. K169, that improved the BAM mineralizing activity of MSH1 in sand microcosms that mimicked the sand filter environment and overruled negative effects from other highly antagonistic sand filter resident bacteria within artificially assembled high-richness communities (9, 10). This so-called benefactor strain apparently engaged in a cooperative interaction with MSH1 by supporting MSH1's BAM mineralization activity. Phylogenetically, *Piscinibacter* sp. K169 relates most to *Piscinibacter aquaticus*, an organism that thrives in oligotrophic waters (11), indicating its adaptation to oligotrophy and that it might easily establish in its habitat of origin (i.e., the sand filter environment). Interestingly, the presence of MSH1 increased the cell densities of K169 in the sand filter microcosms, suggesting that the cooperative interaction between the two strains extends toward a facultative mutualistic interaction from which both strains benefit (i.e., K169 positively affects BAM mineralization in MSH1, while MSH1 increases the cell densities of K169) (9). While the exact mechanism of the interaction between MSH1 and benefactor K169 is not known, its apparent cooperative character might result in simultaneous local micro-scale growth of the two strains, which would further support the co-existence between the two partners by tying together their fitness, expanding their niche, and creating spatial separation from competitors (12, 13).

To introduce and establish an interspecies co-culture system as the MSH1/K169 partnership into the target system, suitable inoculum densities of the consortium partners and suitable growth conditions have to be identified. Initial population sizes of both partners and their ratios are expected to affect the final community structure and, hence, the interactions between the partner organisms (14), for instance, by affecting the exchange efficiency of nutrients, co-factors, and signal molecules, which are main mechanisms of cooperative interactions (15–19). Experimental studies that examine this, though, are scarce and rarely systematically implemented on environmental biotechnology relevant cooperative bacterial partnerships (18, 20, 21). Similarly, theoretical modeling as well as experimental studies suggest that interactions, either synergistic or competitive, between two microbial species can be indirectly mediated and affected by niche conditions determined by diverse environmental factors, including abiotic (e.g., nutrients including carbon sources, toxins, temperature, pH, and oxygen) and biotic (e.g., bacteria, plants, and herbivores) factors (22–26). However, again, experimental studies focusing on environmental cooperative consortia and especially those of biotechnological relevance are rare (18, 27–29). In case of the MSH1/K169 partnership, the available organic carbon sources in the sand filter microcosms provide niches for both strains, so any supplemented substrate like acetate that was externally added in previous studies to the sand filter microcosm as well as the carbon source intrinsically present on and provided by the sand might play a role in the interaction between MSH1 and K169. As such, in order to apply the “co-inoculation” concept, such as MSH1 combined with K169, in a real bioaugmentation context, identifying ideal inoculum densities as well as identifying the carbon sources that drive the cooperation are key prerequisites.

This study aims to acquire an understanding of (i) the constraints for a successful cooperation between MSH1 and K169, in particular, the initial cell densities of MSH1 and K169 and (ii) the environmental factors that govern the cooperative interaction between MSH1 and K169 focusing on the role of carbon/energy resources. To this end, mono- and dual-species sand filter microcosm experiments were performed as described (9) but with modified and varying conditions. In the first two experiments, we varied initial cell densities of one or both strains to examine the effect of cell density ratio and cell density. In two other experiments, we examined the role of the carbon source. We first varied the concentration of acetate externally added in earlier experiments as a carbon source. In a second experiment, we examined the role of intrinsic carbon present on the sand used in the experimental set-up.

## MATERIALS AND METHODS

### Bacterial strains and culture conditions

MSH1-GFP, a GFP-labeled variant of *A. niigataensis* MSH1 carrying the GFP-2X-miniTn5Km gene cassette, was used (8). MSH1-GFP was grown from a cryostock on R2A (R2B [30] containing agar) amended with 100 mg/L BAM and 50 mg/L kanamycin for 7 days. A smear of colonies was then transferred to 50 mL R2B amended with 10 mg/L BAM and incubated for 2 days at 25°C. A rifampicin-resistant variant of *Piscinibacter* sp. K169 (i.e., K169-RIF) (9) was used. K169-RIF was grown from a cryostock on R2A for 7 days, and a colony was transferred into 50 mL R2B and grown for 3 days at 25°C.

### Microcosm set-up

Fig. S1.1 shows a schematic overview of the used microcosm set-up and experimental design. Standard microcosm experiments were performed in 96-well deep plates (Thermo Scientific Nunc, Waltham, MA, USA) containing 150 mg washed autoclaved sand (1–2 mm particle size, Sibelco Benelux, Brussels, Belgium) as described previously (9). Briefly, the sand in the wells was inoculated with 100 µL bacterial culture washed and resuspended in the minimal medium MMO (31) containing 150 µg/L sodium-acetate at a cell density of 10^7^ cells/mL (for each strain) as either mono-species (*R_T_* = 1) or dual-species (*R_T_* = 2) systems with four replicates. After the so-called competition phase (i.e., 7 days of incubation at 20°C [t_7_]), 5,000 counts per minute (cpm) [ring-U−^14^C]-labeled BAM (Institute of Isotopes Co., Ltd., Budapest, Hungary) was added to determine BAM mineralization. Identically treated sand filter microcosms in 96-well deep plates were set up simultaneously (four replicates) to determine MSH1 and K169 cell densities. Sterile abiotic controls, that is, microcosms without bacteria, were included in each experiment. Microcosms to examine the effect of different initial MSH1/K169 cell densities and cell density ratios were set up equally as the standard microcosms but with different cell densities of MSH1 and K169 nominally ranging between 10^7^ and 10^3^ cells/mL (in quadruplicate) (see Fig. S1.1). The used nominal and actual cell densities (as determined by flow cytometry) and ratios are shown in Table S1.1. To test the effect of added acetate concentrations, as in the standard condition, the sand in the wells were inoculated with 100 µL of a bacterial suspension in MMO medium at a cell density of 10^7^ cells/mL (for each strain) but varying concentrations of sodium acetate were added (i.e., 0, 50, and 100 µg/L) in addition to the standard concentration of 150 µg/L (Fig. S1.1). For each acetate concentration, the dual-species assembly MSH1 + K169 and the mono-species systems containing only MSH1 or K169 were tested (in quadruplicate). To reveal the role of sand matrix and organic carbon present on the sand, quadruplicate microcosms were set up similar to the standard microcosms but with and without sand matrix, with sand devoid of organic carbon, and with organic carbon extracted from the sand (Fig. S1.1; Table S1.2). No acetate was added. Sand devoid of organic carbon was obtained by muffling the sand at 550°C for 5 h in a muffle furnace (Nabertherm L9/11/B410, Lilienthal, Germany). Organic carbon from the sand was extracted by mixing 30 g of washed autoclaved sand and 20 mL of MMO medium in muffled glass vials for 8 h at 50 rpm on a test-tube rotator (Labinco L29, Breda, Netherlands). The dissolved organic carbon (DOC) extract was separated from the sand grains by filtration over a 0.45 µm filter (Chromafil PET 0.45 mm, 25 mm diam). The DOC extract contained 37.1 mg C/L of organic carbon as determined using a TOC analyzer (Shimadzu, TOC-L) and was used to resuspend the K169/MSH1 cells for inoculation in wells without and with muffled sand. BAM mineralization and cell densities at t_7_ were determined as described (9). BAM mineralization was followed for 130 h at 19 time points (0.5, 1, 1.5, 2, 2.5, 3, 3.5, 4, 4.5, 5, 7, 8, 9, 11, 12, 16, 26, 55, and 130 h), and the cumulative percentage ^14^CO_2_ relative to the total amount of ^14^C added was plotted as a function of incubation time. BAM mineralization kinetic parameters *λ*, *µ*, and *A* were obtained from the curves using the modified Gompertz model (32), that is,


$$P\left(t\right)=A\;\;exp\left(-exp\left(\frac{e}{A}\left(\mu \lambda -\left(\mu -c\right)t\right)+1\right)\right)+\;ct$$

in which *P(t*) (%) refers to the percentage mineralization at time *t* (h); *A* (%) is the final total extent of mineralization; *λ* (h) is the lag time*; µ* (% h^−1^) is the maximum mineralization rate constant; and *c* (% h^−1^) is the endogenous mineralization rate constant. Kinetic parameters were determined by using the Lsqnonlin command in MATLAB R2012b (Mathworks, Natick, MA, USA) as described (9). Parameter *c* was always close to zero. As done before (9), *λ* and *µ* were used to score the effects of K169 on MSH1 and the nature of the interactions (positive, neutral, or negative) in the direction of K169 toward MSH1. Significantly (95% significance level) decreased *λ* and/or increased *µ* in the dual species systems compared to the MSH1 mono-species system pointed to positive effects, while significantly increased *λ* and/or decreased *µ* scored as negative interactions. No effects on *λ* and *µ* were defined as neutral interactions. *A* was not used for that purpose since a fraction of BAM-derived carbon will be assimilated into biomass in addition to being released as CO₂, and this proportion can change according to the conditions. Moreover, in treatment systems with a continuous water flow like sand filters, the effectiveness of bioaugmentation depends on the degrader’s capacity to grow and establish rapidly, which is captured by *λ* and *μ* and not by *A* (9). The cell densities of MSH1 (*D_MSH1_*) and K169 (*D_K169_*) at t_7_ were determined by extracting the cells from the microcosms as described (9) and counting colony-forming units (CFU) by drop plating 10 µL of 10-fold dilution series on selective agar media (R2A + 100 mg/L BAM containing 50 mg/L Km for selectively counting MSH1 and R2A containing 50 mg/L rifampicin for selectively counting K169). Moreover, CFU on the selective agar medium R2A + 100 mg/L BAM + 50 mg/L Km were checked for GFP fluorescence on a blue light transilluminator to confirm the identity of the colonies as MSH1.

### Determination of organic carbon desorption from sand

Organic carbon desorption from sand was performed in triplicate by adding 30 g autoclaved sand and 20 mL MMO medium in muffled 50 mL glass vials. The vials were incubated statically at 20°C for 7 days. Every 24 h, the solution was filtered over a 0.45 mm filter (Chromafil PET 0.45 mm, 25 mm diam) and replaced by 20 mL MMO medium, except for three vials of which the solution was only filtered at day 7. The DOC content in all extracts was determined as described above.

### Statistics

Significant differences (*P* < 0.05) between *λ*, *µ*, *A*, *D_MSH1_*, and *D_K169_* in different assemblies and conditions were determined with pairwise Tukey test using IBM SPSS Statistics 22.

## RESULTS

### Impact of initial MSH1 and K169 cell densities

**Fig 1:**
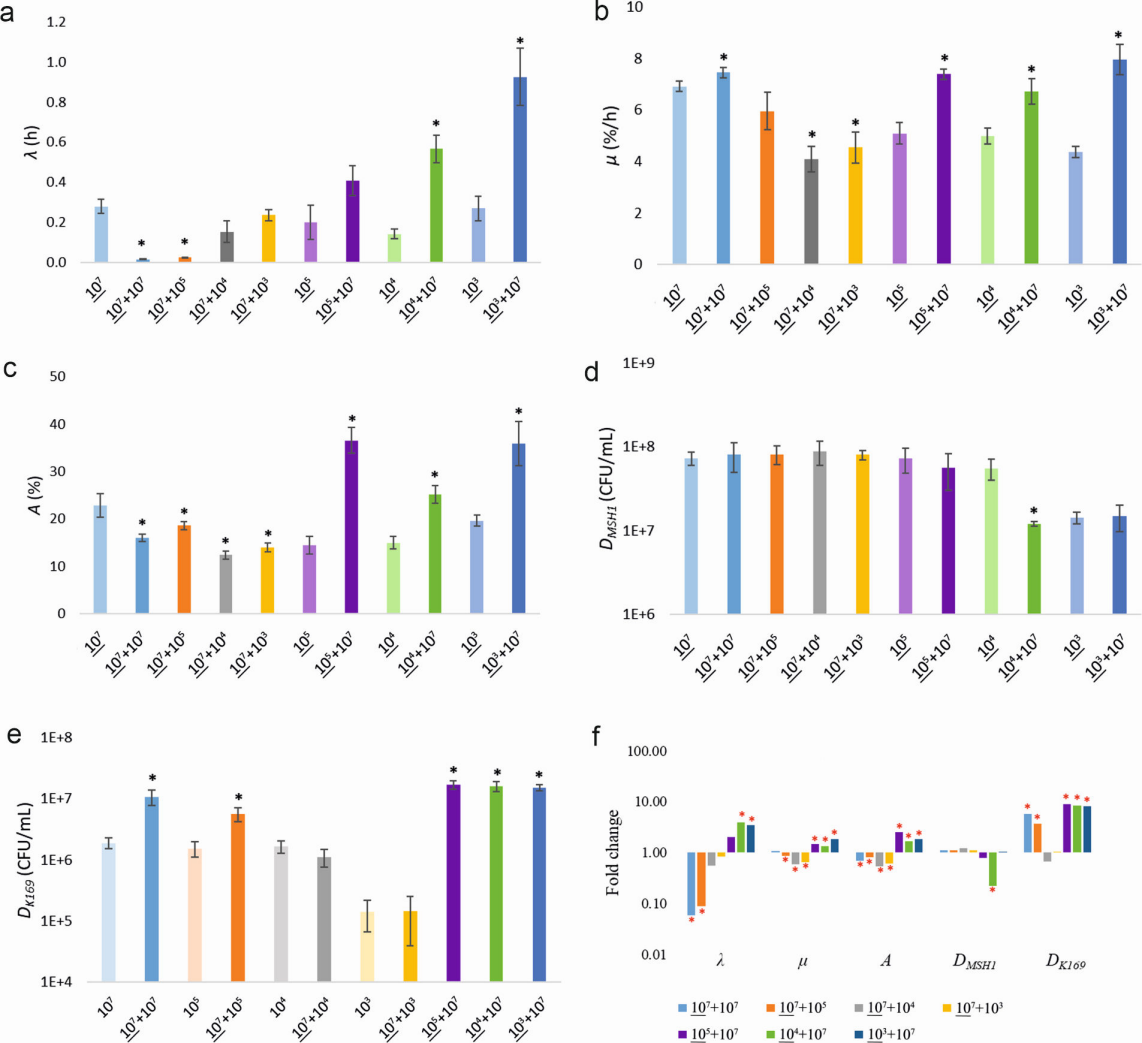
Lag time *λ* (**a**), maximum rate *µ* (**b**), and extent *A* (**c**) of BAM mineralization by MSH1, MSH1 cell densitiy *D_MSH1_* (**d**) and K169 cell density *D_K169_* (**e**) at the end of the competition phase at t_7_, and fold change of the respective parameters between dual-species (*R_T_* = 2) and corresponding mono-species (*R_T_* = 1) systems (**f**) in sand microcosms inoculated with different initial cell densities of either MSH1 or K169. The numbers in the *X*-axis (**a–e**) or under the graph (**f**) indicate the nominal starting cell densities of MSH1 and/or K169. Used initial cell densities of MSH1 are underlined. The values shown are averages with standard deviation (shown in error bar) based on four replicates. The black asterisk (**a to e**) indicates values that significantly differ between *R_T_* = 2 and *R_T_* = 1 systems in microcosms with the same initial MSH1 and K169 cell density (*P* value < 0.05). The red asterisk in (**f**) indicates values for which the difference between *R_T_* = 2 and *R_T_* = 1 systems was significant (*P* value < 0.05). The color of each bar corresponds to those used for indicating the mineralization curves in Fig. S2.1 and S2.2 from which the respective parameter values were deduced.

To evaluate whether MSH1 or K169 requires a certain initial cell density for the cooperative interaction to take place, the effect of different initial cell densities of either K169 or MSH1 combined with a fixed cell density of MSH1 or K169, respectively, leading to different initial MSH1/K169 ratios, was tested. Mineralization kinetics are shown in Fig. S2.1 and S2.2. Values of *λ*, *µ*, *A*, *D_MSH1_* , and *D_K169_* are shown in Table S2.1 and graphically presented in Fig. 1a through e. Calculated fold changes of the respective parameters between dual-species systems and relevant mono-species systems are shown in Table S2.2; Fig. 1f. As reported previously (9), when MSH1 and K169 were co-inoculated at equal starting densities of 10⁷ cells/mL, the presence of K169 enhanced BAM mineralization, as indicated by a shorter *λ* and a higher *μ*. At the same high initial cell density of MSH1 (10^7^ cells/mL) but decreasing initial cell densities of K169 (from 10^7^ to 10^3^ cells/mL), the positive effect of K169 on BAM mineralization turned into a negative effect. The positive effect on *λ* (especially at 10^4^ cells/mL and lower) gradually disappeared until it reached 0.24 h, which is not significantly different from the value observed in the MSH1 mono-species system (0.28 h) (Fig. 1a and f ). Similarly, *µ* values reduced until 4.54%/h, which is significantly lower than this of the MSH1 mono-species (6.92%/h) when K169 initial cell densities of 10^4^ cells/mL and lower were applied, indicating negative effects (Fig. 1b and f). *A* values decreased with decreasing K169 cell densities (Fig. 1c and f). Despite the effect on the BAM mineralization kinetics, *D_MSH1_* in the dual-species systems was not affected (Fig. 1d and f). On the other hand, *D_K169_* in the dual-species systems decreased with decreasing initial K169 cell densities. Moreover, *D_K169_* did reach similar but not higher values compared to those in the corresponding K169 mono-species when the inoculated K169 cell density was 10^4^ cells/mL and lower, suggesting loss of the benefit from MSH1 for K169 at lower initial K169 densities (Fig. 1e and f). We conclude that reducing initial K169 cell densities has a negative effect on the cooperation between MSH1 and K169 by impacting both the BAM mineralization kinetics and growth of K169 negatively, especially at K169 cell densities of 10^4^ cells/mL and lower.

**Fig 2:**
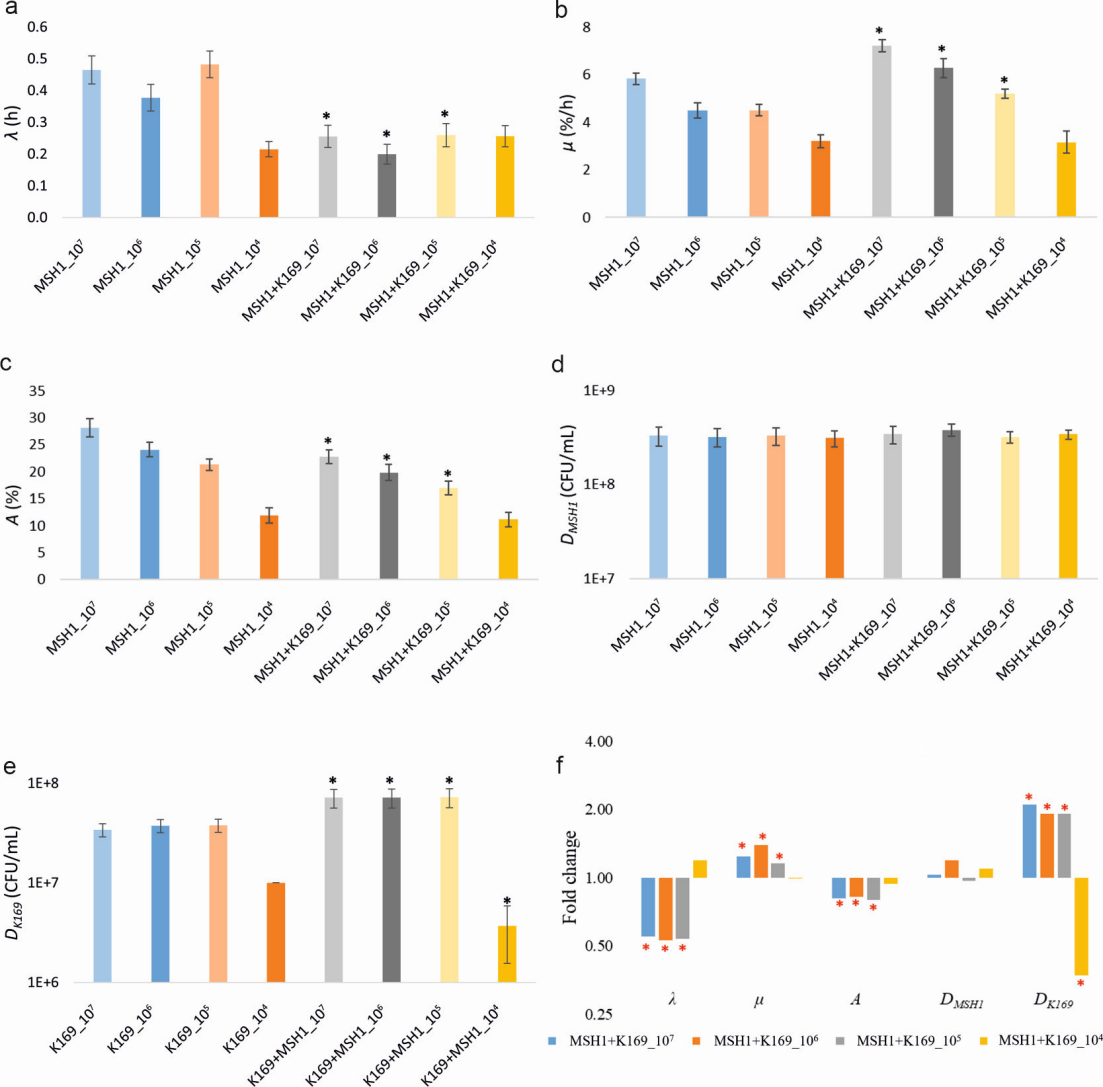
Lag time *λ* (**a**), maximum rate *µ* (**b**), and extent *A* (**c**) of BAM mineralization by MSH1, MSH1 cell density *D_MSH1_* (**d**) and K169 cell density *D_K169_* (**e**) at the end of the competition phase at t_7_, and fold change between the respective parameter in dual-species assembly (*R_T_* = 2) and relevant mono-species systems (*R_T_* = 1) (**f**) in sand microcosms containing *R_T_* = 2 systems with identical starting cell densities of MSH1 and K169 from 10^7^ to 10^4^ cells/mL and of corresponding *R_T_* = 1 systems of the two strains. The numbers in the *X*-axis (**a–e**) or under the graph (**f**) indicate the nominal starting cell densities of MSH1 and/or K169. The values shown are averages with standard deviation (shown in error bar) based on four replicates. The black asterisk (**a to e**) indicates values that significantly differ between *R_T_* = 2 and *R_T_* = 1 systems in microcosms with the same starting MSH1 and K169 cell densities (*P* value < 0.05). The red asterisk in (**f**) indicates values for which the difference between *R_T_* = 2 and *R_T_* = 1 systems was significant (*P* value < 0.05). The color of each bar corresponds to those used for indicating the mineralization curves in Fig. S2.3 from which the respective parameter values were deduced.

At a constant initial cell density of K169 of 10^7^ cells/mL, decreasing the initial MSH1 cell density from 10^7^ to 10^3^ cells/mL clearly affected BAM mineralization as *λ* increased although *µ* remained unchanged (Fig. 1a and b). Interestingly, comparing the MSH1 + K169 assemblies with the respective MSH1 mono-species systems at the same initial cell densities, it can be observed that (i) the positive effect on *λ* observed at 10^7^ cells/mL turned into a neutral or even negative effect at MSH1 cell densities of 10^5^ cells/mL or lower while (ii) the positive effect on *µ* remained (Fig. 1a, b and f). Besides, *A* values also changed from lower values compared to MSH1 mono-species at 10^7^ MSH1 cells/mL to higher values compared to MSH1 mono-species at 10^5^ MSH1 cells/mL and lower (Fig. 1c and f). The change in BAM mineralization kinetic parameters at lower initial cell densities of MSH1 was congruent with lower MSH1 cell densities at t_7_ as shown by the *D_MSH1_* values in the dual-species systems (Fig. 1d). However, the low MSH1 cell densities at t_7_ cannot be the only explanation; likely also altered K169-MSH1 interactions contributed since *D_MSH1_* in the MSH1 + K169 combination inoculated with 10^4^ MSH1 cells/mL was 4.6-fold lower than in the MSH1 mono-species system inoculated with 10^4^ MSH1 cells/mL, though this was not observed in the case of initial MSH1 cell densities of 10^3^ MSH1 cells/mL (Fig. 1d and f). K169 cell densities were not impacted (Fig. 2e). We conclude that decreasing the initial MSH1 cell densities in the dual species systems negatively impacted the cooperation between MSH1 and K169 at MSH1 cell densities of 10^4^ cells/mL and lower, especially impacting BAM mineralization, while growth of K169 was not impacted.

We then tested the impact of decreasing initial but identical cell densities of both strains (i.e., 10^7^–10^4^ cells/mL) in a second experiment. Graphs are shown in Fig. S2.3. Values of *λ*, *µ*, *A*, *D_MSH1_*, and *D_K169_* are shown in Table S2.3 and in Fig. 2a through e. Calculated fold changes of the respective parameters between dual-species and respective mono-species systems are shown in Table S2.4; Fig. 2f. Despite MSH1 showing the same cell density at t_7_ (3.1–3.8 × 10^8^ CFU/mL), BAM mineralization kinetics differed substantially, especially regarding *µ*. At initial cell densities of 10^7^, 10^6^, and 10^5^ cells/mL, *λ* and *µ* remained respectively lower and higher compared to their values in the corresponding MSH1 mono-species systems, and *λ* was even lower compared to the mono-species system with initial densities of 10^7^ cells/mL (Fig. 2 a and b). *A* values declined as cell density decreased. At initial cell densities of 10⁷, 10⁶, and 10⁵ cells/mL, *A* was lower than in the corresponding MSH1 mono-species systems (Fig. 2c; Table S2.3). However, at initial cell densities of 10^4^ cells/mL, no beneficial effect of K169 was observed compared to the MSH1 mono-species systems. Similarly, *D_K169_* values were independent of the initial cell densities and improved when MSH1 was present (factor 1.9–2.1), except at initial cell densities of 10^4^ cells/mL where K169 did reach lower numbers and appeared to be negatively affected by MSH1 (factor 2.7). These results show that the beneficial effects of K169 on MSH1, and vice versa, and, hence, the cooperation also occur when both strains are inoculated at cell densities as low as 10^5^ cells/mL. However, at lower cell densities, the cooperation deteriorated.

### Impact of acetate concentration

**Fig 3:**
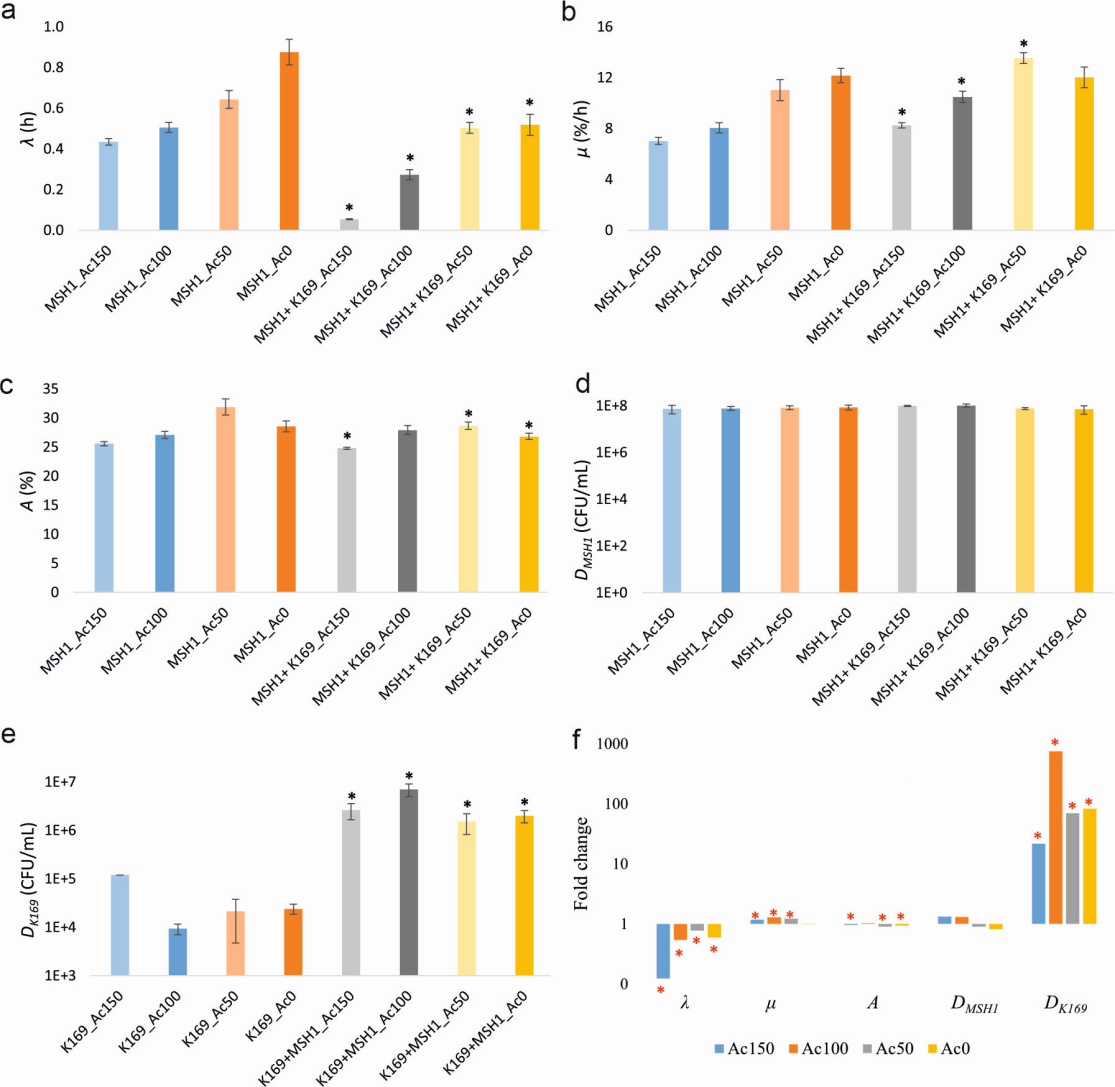
Values of BAM mineralization kinetic parameters *λ* (**a**), *µ* (**b**), and *A* (**c**), MSH1 cell density *D_MSH1_* (**d**) and K169 cell density *D_K169_* (**e**), and fold change of the respective parameters between dual-species (*R_T_* = 2) and corresponding mono-species (*R_T_* = 1) systems (**f**) at t_7_ in sand microcosms amended with different concentrations of acetate (Ac). Each microcosm has identical starting densities of MSH1 and K169 of 10⁷ cells/mL for each strain. The numbers in the axis (**a–e**) or under the graph (**f**) indicate the applied acetate concentration. The values shown are averages with standard deviation (shown in error bar) based on four replicates. The black asterisk (**a to e**) indicates values that significantly differ between *R_T_* = 2 and *R_T_* = 1 systems in microcosms with the same acetate concentration (*P* value < 0.05). The red asterisk (**f**) indicates values for which the difference between *R_T_* = 2 and *R_T_* = 1 systems was significant (*P* value < 0.05). The color of each bar corresponds to those used for indicating the mineralization curves in Fig. S3.1 from which the respective parameter values were deduced.

Different concentrations of sodium acetate (i.e., 0, 50, 100, and 150 µg/L) were tested to examine the effect on the MSH1–K169 interaction. Both strains were inoculated at 10^7^ cells/mL. Values of *λ*, *µ*, *A*, *D_MSH1_*, and *D_K169_* are shown in Table S3.1 and Fig. 3a through e. Calculated fold changes of the respective parameters between dual-species and respective mono-species systems are shown in Table S3.2; Fig. 3f. Decreasing acetate concentrations resulted in increasing *λ* and *µ*, taking the standard situation (150 µg/L sodium acetate) as a reference (Fig. S3.1; Table S3.1). However, for most acetate concentrations, the values of *λ* and *µ* remained respectively lower and higher in the dual-species systems compared to the MSH1 mono-species systems amended with the same acetate concentration (Fig. 3a and b). The exception was when no acetate was added where *λ* was still lower in the dual-species system compared to the MSH1 mono-species system, but *µ* became equal. Nevertheless, the fold change for *λ* between dual- and mono-species situations increased with decreasing acetate concentration, indicating that the effect of K169 on BAM mineralization weakened with decreasing acetate concentrations (Fig. 3f). *A* values in dual-species systems were lower than in monocultures, except when the acetate concentration was 100 µg/L. Cell densities of MSH1 (*D_MSH1_*) were not affected, showing equal cell densities in both mono- and dual- species systems independent of the added acetate (Fig. 3d and f). The beneficial effect of MSH1 on K169 growth and final cell densities (*D_K169_*) appeared independent of the acetate concentration, with the density of K169 being 83-fold higher in the presence of MSH1 compared to without MSH1, even when no acetate was supplemented (Fig. 3e and f). We conclude that acetate plays only a moderate role in the positive impact of K169 on MSH1 functionality while being not essential for the positive effect of MSH1 on the growth of K169.

### Impact of sand organic carbon

**Fig 4:**
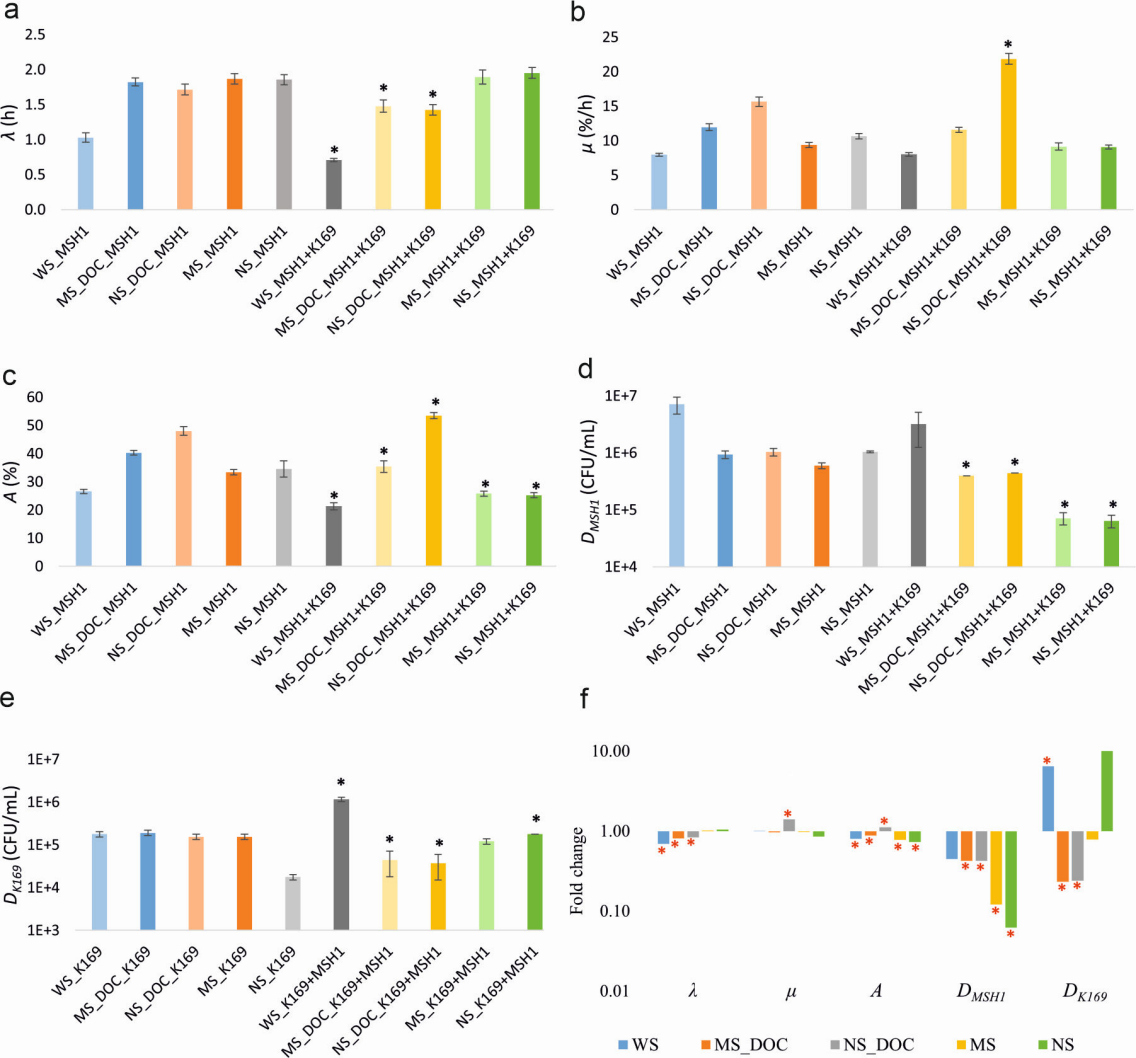
Values of BAM mineralization kinetic parameters *λ* (**a**), *µ* (**b**), and *A* (**c**), MSH1 cell density *D_MSH1_* (**d**) and K169 cell density *D_K169_* (**e**), and fold change of the respective parameters between dual-species (*R_T_* = 2) and corresponding mono-species (*R_T_* = 1) systems (**f**) at t_7_ in sand microcosms with normal sand (WS), muffled sand (MS), and no sand (NS) and with and without amendment of DOC extracted from the sand (DOC: with DOC extract added) to assess the role of sand organic carbon and the sand matrix on the mutualistic interactions between MSH1 and K169. Each microcosm has identical starting densities of MSH1 and K169 of 10⁷ cells/mL for each strain. The values shown are averages with standard deviation (shown in error bar) based on four replicates. The black asterisk (**a to e**) indicates values that significantly differ between *R_T_* = 2 and *R_T_* = 1 systems operated under identical conditions of added sand and DOC (*P* value < 0.05). The red asterisk (**f**) indicates values for which the difference between *R_T_* = 2 and *R_T_* = 1 systems was significant (*P* value < 0.05). The color of each bar corresponds to those used for indicating the mineralization curves in Fig. S4.1 from which the respective parameter values were deduced.

Since acetate plays a minor role in the interactions between MSH1 and K169, another C-source must feed the cooperation. The sand, although washed and autoclaved, might contain assimilable organic carbon. Therefore, the role of the organic carbon present on the sand was examined. No acetate was added in this experiment, and both strains were always inoculated at 10^7^ cells/mL. Mineralization curves are shown in Fig. S4.1. Values of *λ*, *µ*, *A*, *D_MSH1_*, and *D_K169_* are shown in Table S4.1; Fig. 4a through e. Calculated fold changes of the respective parameters between dual-species and respective mono-species systems are shown in Table S4.2; Fig. 4f. Organic carbon on the sand was essential for the cooperation since in microcosms containing sand devoid of organic material (achieved by muffling the sand) or in microcosms without sand, the cooperative interactions disappeared from both partner’s sites. Specifically, *λ* and *µ* values became equal to those in mono-species systems*; D_MSH1_* was lower (8.3-fold in muffled sand, 17-fold in case sand was not added) in the presence of K169 compared to *D_MSH1_* in the MSH1 mono-species system; and *D_K169_* became equal. Moreover, when DOC extracted from the sand was added to muffled sand, the beneficial effect was restored as *λ* lowered in the dual-species system compared to that in the MSH1 mono-species system (respectively 1.48- and 1.82-fold)), while *µ* was equal. However, the densities of MSH1 and K169 were respectively 2.4- and 4.3-fold lower in the dual-species system than in the mono-species system. The role of DOC was emphasized when DOC extracted from sand was added to microcosms without sand, as under those conditions, *λ* was lower and *µ* higher compared to those in the MSH1 mono-species system, pointing to a beneficial effect of K169 on BAM mineralization. However, in the microcosms containing added DOC without sand, *D_MSH1_* and *D_K169_* were lower (respectively 2.3- and 4.1-fold) in the dual-species system compared to the mono-species systems. In addition, *D_K169_* in dual-species systems was lower in microcosms containing added DOC compared to the conditions without added DOC (factor 2.7 in muffled sand and factor 4.8 in the no sand systems). A plausible explanation for the reduced MSH1 and K169 cell densities in systems with DOC extract might be related to the total lower organic carbon content of the DOC extract compared to this in the original sand filter microcosm, as only a fraction of the DOC was likely extracted due to equilibrium between organic carbon absorbed to the sand and dissolved in water. Therefore, we performed a DOC desorption experiment with the original sand and examined whether additional organic carbon could be extracted from the sand after a first equilibrium step. This was indeed the case. Cumulative desorption reached 56.0 mg of organic C/L (Fig. S4.2), while the DOC solution obtained from only one extraction step contained 35.0 mg of organic C/L. We conclude that the organic carbon on the sand is crucial for the cooperation between MSH1 and K169.

## DISCUSSION

### Differences in initial MSH1/K169 cell ratio affect the cooperative interaction between K169 and MSH1

Previously, a cooperative interaction between *A. niigataensis* MSH1 and *Piscinibacter* sp. K169 was observed when MSH1 and K169 were both inoculated at initial cell densities of 10^7^ cells/mL. MSH1 had a beneficial effect on the cell density of K169 by promoting its growth, and vice versa, K169 had a positive effect on BAM mineralization despite not affecting MSH1 cell densities (9). As observed before (9, 10), we noted that the parameters of kinetics of BAM mineralization, *D_MSH1_* and *D_K169_*, both in mono-culture and dual culture systems varied between experiments both in this study and compared to experiments in the earlier studies despite the high degree of standardization in setting up the experiments. The mechanisms underlying this variability are currently unclear. Nevertheless, the positive interaction and apparent cooperation between both strains within the same experiment were always significant and obvious when MSH1 and K169 were both inoculated at initial cell densities of 10^7^ cells/mL. The mechanism of this cooperation is presently unknown but likely follows other bacterial cooperative interactions explained by the (i) removal of waste products hampering the functionality of the partner (33, 34), (ii) exchange of growth substrates or compounds like co-factors and vitamins (13, 15, 19, 35), and (iii) inter-species chemical signaling (17, 36).

Altering the initial inoculum densities impacted community structure as well as the interactions between the two partners, resuting in impacted BAM mineralizing functionality, albeit only when the initial cell densities of one of the partners, either MSH1, K169 or both, were relatively low. It supports the study from Gao et al. (18), which concluded that community assembly and interactions are not intrinsic properties of the two species but are emergent properties that can be regulated in response to the initial relative abundances of the involved species. Similarly, Aharonovich and Sher (20) and Venturelli et al. (21) reported that the initial ratio altered the nature of interspecies interactions, while Katsuyama et al. (14) predicted that the dynamics and co-existence of two members of a pesticide-degrading interspecies consortium depended on the relative initial population sizes next to their reciprocal activities that governed the cooperation. As observed by Gao et al. (18), alterations in the interactions are mostly observed when one of the two members is relatively low in initial density. Low K169 or low MSH1 cell numbers might have resulted in a failure to establish the metabolic coupling pathway between both organisms required for cooperation (18), for instance, by insufficient turnover of waste products or poor production of the compounds that promote MSH1 functionality and K169 growth. Interestingly, in the case of low initial K169/MSH1 ratios, the MSH1 functionality was affected and not *D_MSH1_*, supporting the hypothesis that K169 mainly supports the MSH1 activity (9). Another feature of interest was that in the case of high initial MSH1/K169 ratios and high initial K169/MSH1 ratios, *D_K169_* and *D_MSH1_* were lower than the final densities observed for their respective mono-culture systems. This might be explained by competition for growth resources between the two organisms. As shown in this study, the organic carbon on the sand represents a major C-source for the partnership. While the exact composition of the organic carbon on the sand is currently unknown, it likely consists of different compounds of which a fraction might be shared between the partners (37). Possibly, when one of the two organisms is initially present at much higher cell densities than the other, the former might acquire a better share of the available communal sand carbon sources. However, neither the poor establishment of metabolic coupling nor competition for resources is expected when both organisms are present at low initial cell densities of 10^4^/mL, while under these conditions, the partnership was negatively affected. Therefore, we propose that ideal spatial patterns for efficient cooperation might have been impacted at lower cell densities as earlier observed for interactions between producers of public goods and cheats (38–40) (i.e., when MSH1 cells and K169 are inoculated both at rather low cell densities, the two partners grow but segregated from each other so that the interactions are limited). The availability of the sand particles as substratum for colonization might have contributed to that spatial effect. Overall, we hypothesize that the observed negative impact of decreasing inoculum cell densities, especially at cell densities of 10^4^ cells/mL and lower, on the cooperation (i.e., MSH1 functionality and final K169 cell densities) relates to a complex interplay of competition, spatial features, and non-successful establishment of the required metabolic coupling pathways. A corresponding plausible but yet hypothetical scenario is illustrated in Fig. S5.

With respect to the application of partnership for the bioaugmentation of DWTP sand filters, it can be suggested that inoculation with similar cell densities of both strains is recommended but that relatively low cell densities can be used, for instance, 10^5^ cells/mL, compared with those that were initially applied to identify the cooperation (i.e., 10^7^ cells/mL) of each partner. Using less bacterial cells for inoculation is important since culturing the inoculum in dedicated bioreactors contributes largely to the cost and the sustainability of using bioaugmentation of sand filters as a drinking water remediation approach (41). Earlier, Schultz-Jensen et al. (42) proposed an approach for culturing MSH1 until high cell densities in a 5 L bioreactor. However, in field systems, the inocula have to invade and be maintained in an existing biofilm community in a DWTP sand filter. DWTP sand filter microbial communities show high cell density and high diversity (43–45). Both are expected to negatively affect invasion due to exploitative and/or interference competition of the inocula with residents (46). Increasing propagule pressure (i.e., cell densities used at inoculation) has a positive effect on invasion success (47, 48), and it has to be awaited whether the proposed lower inoculum cell densities are sufficient for invasion by MSH1, even with K169 as a support. Previously, MSH1 was shown to successfully invade sand filter resident communities in sand filters at laboratory and pilot scale using inocula with cell densities of 10^7^–10^9^ cells/mL (6, 7, 49), while the addition of K169 was shown to alleviate negative effects of community diversity on MSH1-mediated BAM mineralization in synthetic higher-richness communities (10).

### Organic carbon on sand affected the cooperative interaction

Decreasing the acetate concentration did not affect the cooperative interaction, except for a loss in increase of *µ* value (12.01%/h and 12.17%/h, respectively, in MSH1 mono- and dual-species system) when no acetate was added. We previously showed that this increase in *µ* was not always observed, even when acetate was added at 150 µg/L (9). These observations indicate that acetate is not crucial for the interaction between K169 and MSH1, and that other C-sources likely present on the sand compose the main energy and C-source for driving the cooperative interaction. That the carbon on the sand contains potential C-sources was not unexpected since we previously showed that some sand filter isolates that did not grow on acetate nevertheless proliferated in the sand filter microcosm (9). Moreover, the concentration of organic carbon that was desorbed from the sand in the microcosms (35 mg C/L) was 875-fold higher compared to the supplemented acetate (0.04 mg C/L), identifying the carbon on the sand as the major potential C-source in the system.

Sand in the microcosms and its intrinsic organic carbon was indeed found to be essential for survival and/or growth of MSH1 and K169 and the interactions between them. The cell densities of MSH1 and K169 decreased to levels that were even lower than those at inoculation in systems without sand (NS) and systems containing sand devoid of organic carbon (MS), while the typical promotion of BAM mineralization in the presence of K169 was lost. By contrast, the positive effect of K169 on BAM mineralization was restored when DOC extracted from the sand was added (MS_DOC and NS_DOC microcosms). However, the final cell density of MSH1 in the mono-species systems containing the added DOC extract was lower compared to the MSH1 mono-species system containing the original sand and further decreased in the presence of K169, regardless of the presence of the sand matrix. Similarly, the cell density of K169 was negatively affected by MSH1 in the dual-species system when DOC was added either with or without sand (MS_DOC and NS_DOC). This is unexpected since even in conditions without any carbon (MS), the K169 density in dual-species system was not changed or even increased in NS compared to its mono-species. Therefore, as in the microcosms with the original sand, the DOC extracted from the sand resulted in K169 supported MSH1 functionality, but it unexpectedly turned the neutral or positive effects on growth of MSH1 and K169 to negative effects. A possible explanation is that the DOC in the extract may quantitatively and/or qualitatively not fully represent the available organic carbon present on the sand leading to less growth or a change in ecological niches for one or both strains, for instance, due to a change in the composition of the organic carbon after extraction. Alternatively, while the DOC extract provides all carbon directly available to the bacteria at start of the experiment, the release of potential carbon substrates when present on the sand will be slower, which might lead to slower but similar growth. A DOC desorption experiment indeed suggested that not all DOC present on the sand became directly available upon extraction. The immediate and complete consumption of the DOC in the extract may jeopardize the longer maintenance of both MSH1 and K169 cell densities in contrast to the slow release of DOC from the original sand. The negative effect on the final biomass of MSH1 and K169 in dual-species systems compared to single-species systems might then be linked with competition between the two partners. Nevertheless, and interestingly, despite the lower cell densities of MSH1 and K169, MSH1 still showed improved mineralization when K169 was present. Apparently, the lower K169 cell densities still improved BAM mineralization in MSH1. Studies have shown that even non-growing cells can continue to generate energy needed for any process that is not directly used to synthesize and polymerize biosynthetic precursors, thereby maintaining metabolic activity (50, 51). Metabolic activities do not depend on growth, so the overall metabolic activity of the cell does not decrease proportionally to the growth rate during the period of nutrient limitation, which can explain our observations of improved BAM mineralization despite the lower MSH1/K169 densities in the DOC extract-amended microcosms. Otherwise, cell densities were determined by plating and CFU counting, so it is also plausible that a fraction of the cells entered a ‘viable but not culturable state (VBNC)’ in microcosms amended with the DOC extract. Fida et al. (52) reported that VBNC cells can remain metabolically active, which is consistent with our observation that the BAM mineralization was still improved.

The observation that organic carbon on the sand grains is key for maintaining the cooperation between the two strains and, hence, the MSH1 functionality, is of interest since it would indicate that addition of an extra carbon and energy source is not required. However, it poses questions about the quality, quantity,and finiteness of the organic carbon present in actual sand filters exploited in DWTPs. In contrast to the non-aged sand used in the microcosm experiments, which contain a high amount of DOC on the surface, the organic carbon in DWTP sand filters is expected to originate from the intake water produced from endogenous microbes or originating from decaying biomass. DOC content and quality in groundwaters, the main drinking water resource that carries BAM, are relatively low (53, 54). Moreover, in DWTPs, DOC present in the intake water is likely removed in the aeration step performed prior to sand filtration (45, 55), and the incoming groundwater in sand filters can be considered oligotrophic containing primarily inorganic electron donors (55). However, the turnover of the relatively high and diverse microbial biomass in sand filters, which likely relates to growth and activity of chemolithotrophic organisms utilizing the inorganic electron donors (44), might sustain heterotrophs like K169. Moreover, as in aquifers, microbial production of secondary compounds might support growth and maintenance of heterotrophs (53). Understanding sources and turnover of organic carbon and the mechanisms that sustain microbial networks and biomass in DWTP sand filters will be key for managing the longevity of the cooperation and for further optimizing the co-inoculation approach for improving bioaugmentation.

### Conclusions

We conclude that the cooperative interaction between MSH1 and K169 (i) depends on the initial population sizes of the two partners but only changes strongly at rather low initial cell densities of one of the partners and (ii) is governed by the intrinsic organic carbon present on the sand. These results sustain the concept of co-inoculation of MSH1 and K169 in bioaugmentation efforts to avert BAM pollution for drinking water production since the cooperative interaction between benefactor K169 and MSH1 appears (i) to develop using relatively low inoculum cell densities and (ii) depend on conditions without extraneously supplemented carbon sources. Further research should focus on the role of propagule pressure in invading native sand filter biofilms and on the utilization and longevity of C-sources intrinsic to actual sand filtration systems in waterworks to fuel and sustain the cooperation.

## SUPPLEMENTAL MATERIAL

Supplemental material aem.01149-25-s0001.docx Additional experimental details and supplemental figures and tables.

ASM does not own the copyrights to Supplemental Material that may be linked to, or accessed through, an article. The authors have granted ASM a non-exclusive, world-wide license to publish the Supplemental Material files. Please contact the corresponding author directly for reuse.
