# Supplementary material for: Boundary conditions for exploiting the cooperation of Aminobacter niigataensis MSH1 with Piscinibacter sp. K169 to support 2,6-dichlorobenzamide biodegradation in sand filters for drinking water treatment: role of cell density and organic carbon
Source: Appl Environ Microbiol. 2025 Sep 25;91(10):e01149-25. doi: 10.1128/aem.01149-25 (PMC12542637; doi:10.1128/aem.01149-25)
Supplement: Supplemental material — Additional experimental details and supplemental figures and tables. [file aem.01149-25-s0001.docx]

**Document S1. Materials and methods**


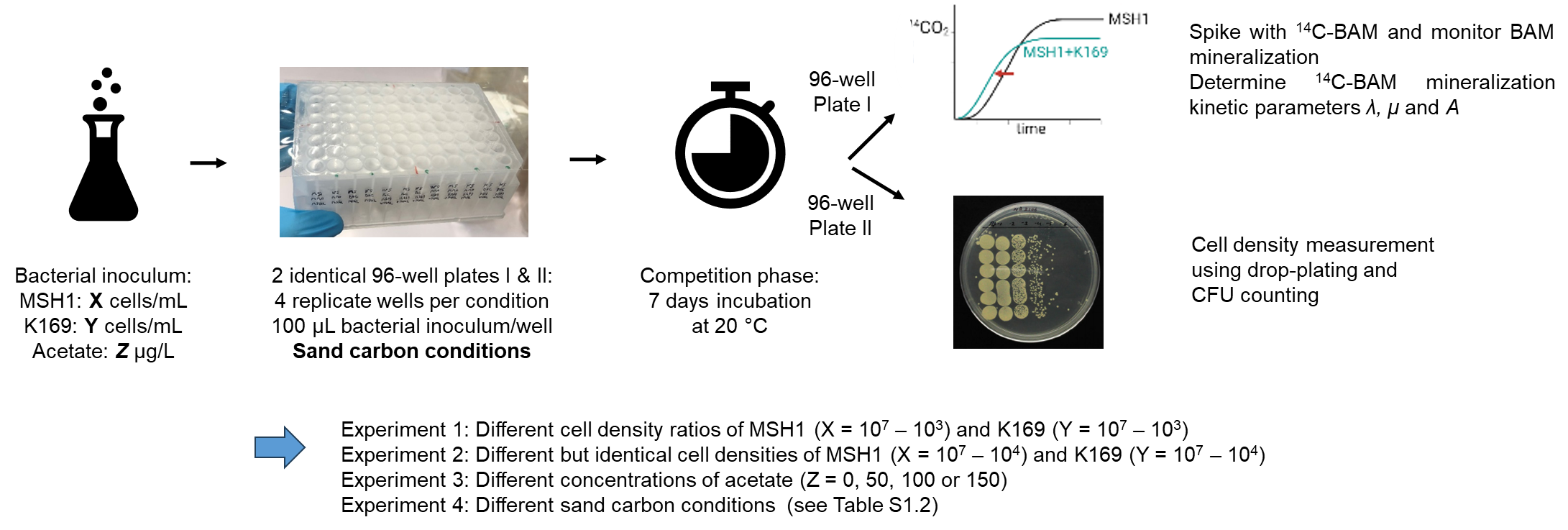


Figure S1.1. Setup and overall workflow used in the sand filter microcosm experiments performed in this study reporting the variables (in bold) that are studied in each experiment according to their order of appearance in the main text. Details are provided in the Materials and Methods.

Table S1.1. Nominal and measured inoculated (initial) cell densities of MSH1 and K169 and corresponding ratios used in the microcosm experiment for testing the effect of different initial MSH1 and K169 cell densities. The actual values are live cell counts of diluted washed cultures determined by Flow Cytometry at day 0.

| **Nominal values** | | | **Actual values** | | |
| --- | --- | --- | --- | --- | --- |
| **Cell density of MSH1 (cells/mL)** | **Cell density of K169 (cells/mL)** | **MSH1/K169** | **Cell density of MSH1 (CFU/mL)** | **Cell density of K169 (CFU/mL)** | **MSH1/K169** |
| 10^7^ | 10^7^ | 1 | 7.72*10^6^ | 5.46*10^6^ | 1.41 |
| 10^7^ | 10^5^ | 100 | 7.72*10^6^ | 7.50*10^4^ | 103 |
| 10^7^ | 10^4^ | 1000 | 7.72*10^6^ | 6.25*10^3^ | 1235 |
| 10^7^ | 10^3^ | 10000 | 7.72*10^6^ | 6.94*10^2^ | 11111 |
| 10^5^ | 10^7^ | 0.01 | 6.15*10^4^ | 5.46*10^6^ | 0.01 |
| 10^4^ | 10^7^ | 0.001 | 5.59*10^3^ | 5.46*10^6^ | 0.001 |
| 10^3^ | 10^7^ | 0.0001 | 6.21*10^2^ | 5.46*10^6^ | 0.0001 |

Table S1.2. Overview of the experimental design to assess the role of organic carbon present on the sand on the cooperative interactions between MSH1 and K169 in microcosms.

|  | **With sand matrix (Designation)** | **Without sand matrix (Designation)** |
| --- | --- | --- |
| **With sand organic carbon** | MMO + sand (WS);  DOC extract in MMO + muffled sand (MS_DOC) | DOC extract in MMO without sand (NS_DOC) |
| **Without sand organic carbon** | MMO + muffled sand (MS) | MMO without sand (NS) |

**Document S2.** **Impact of initial MSH1 and K169 cell densities**


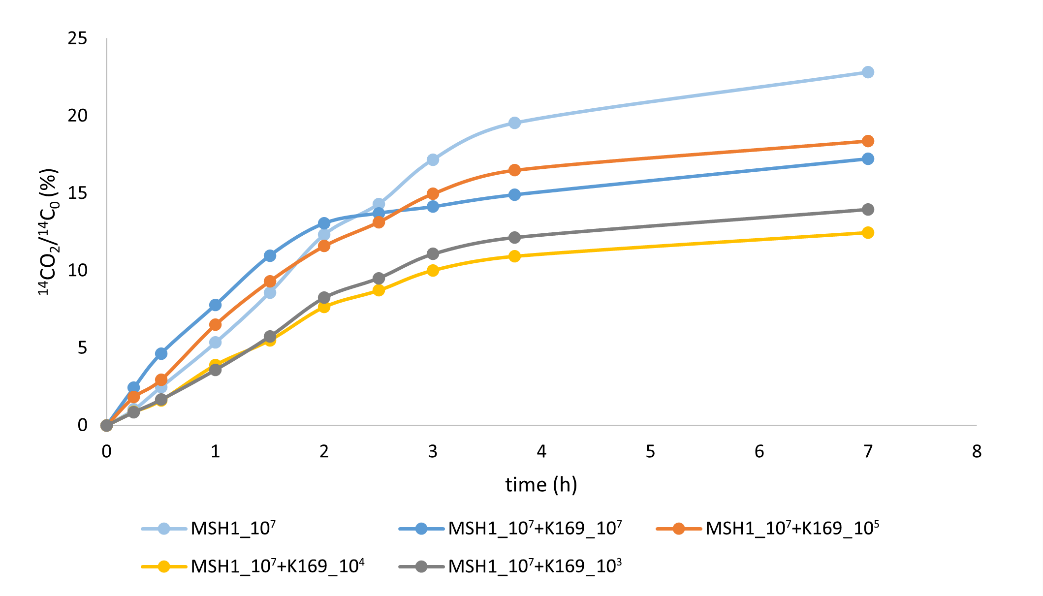


Figure S2.1. Cumulative BAM mineralization curves at t_7_ (measured cumulative ^14^CO_2_ production relative to the initial amount of ^14^C-BAM added (^14^C_0_)) obtained for (i) dual-species systems of *A.* *niigataensis* MSH1 and *Piscinibacter* sp. K169 (*R_T_* = 2) with MSH1 at initial cell densities of 10^7^ cells/mL and decreasing initial cell densities of K169 and (ii) MSH1 in mono-species systems (*R_T_* = 1) at an initial cell density of 10^7^ cells/mL. The different colors refer to the strain combinations as indicated below the graph. The numbers mentioned after the strain names, as indicated below the graph, refer to the starting cell densities at day 0. Values are averages of four replicates. Standard deviations are not shown for clarity but never exceeded 0.4%. The color of each curve corresponds to the bar under the same condition in Figure 1.

| 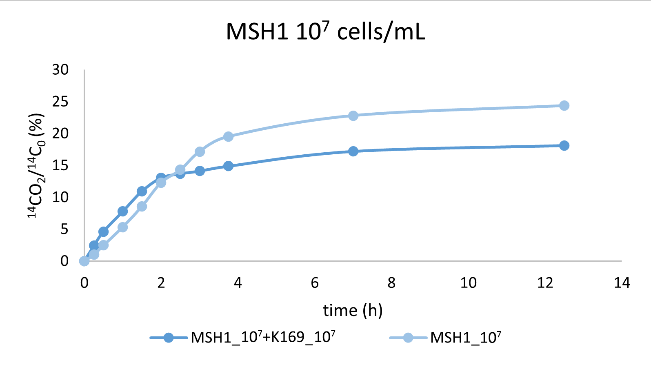  a | 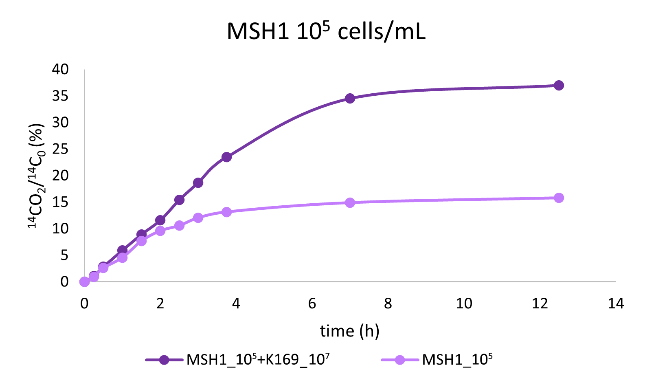  d  b |
| --- | --- |
| 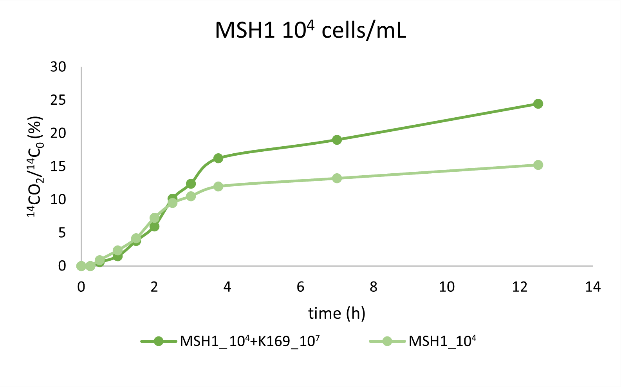  c | 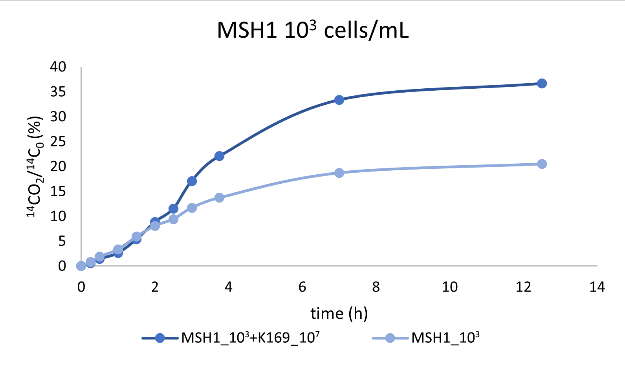 |

Figure S2.2. Cumulative BAM mineralization curves at day 7 (measured cumulative ^14^CO_2_ production relative to the initial amount of ^14^C-BAM added (^14^C_0_)) obtained for dual-species systems of *A. niigataensis* MSH1 and *Piscinibacter* sp. K169 (*R_T_* = 2) at different initial cell ratios and for corresponding MSH1 mono-species systems (*R_T_* = 1) with fixed initial cell density of K169 at 10^7^ cells/mL and initial cell densities of MSH1 of either 10^7^ cells/mL (panel a), 10^5^ cells/mL (panel b), 10^4^ cells/mL (panel c) or 10^3^ cells/mL (panel d). The different colors refer to the strain combinations as indicated below the graph. The numbers after the strain names refer to the initial cell densities of that strain at day 0. Values are averages of four replicates. Standard deviations are not shown for clarity but never exceeded 0.3%. The color of each curve corresponds to the bar under the same condition in main text Figure 1.

Table S2.1. Values of the BAM mineralization parameters and cell densities of MSH1/K169 obtained for dual-species systems of MSH1 and K169 (*R_T_* = 2) and for MSH1 or K169 in mono-species systems (*R_T_* = 1) for different MSH1/K169 initial cell densities. The number after the strains refers to the nominal initial cell densities at day 0. The green numbers indicate a significant positive effect of K169 on the BAM mineralization parameter/cell densities while the red numbers indicate a significant negative effect of K169. The black asterisk indicates parameters that significantly differ (p value < 0.05) between dual-species (*R_T_* = 2) and mono-species (*R_T_* = 1) systems for a certain initial cell density. Average values ± standard deviation of four replicates are given.

|  | ***λ* (h)** | ***µ* (%/h)** | ***A* (%)** | ***D_MSH1_* (CFU/mL)** | ***D_K169_* (CFU/mL)** |
| --- | --- | --- | --- | --- | --- |
| **MSH1_10^7^** | 0.28 ± 0.036 | 6.92 ± 0.20 | 22.80 ± 2.49 | 7.3^E+7^ ± 1.3^E+7^ | \ |
| **K169_10^7^** | \ | \ | \ | \ | 1.9^E+6^ ± 3.8^E+5^ |
| **MSH1_10^7^+K169_10^7^** | 0.02 ± 0.001* | 7.44 ± 0.19* | 15.98 ± 0.81* | 8.0^E+7^ ± 3.1^E+7^ | 1.1^E+7^ ± 3.1^E+6^* |
| **K169_10^5^** | \ | \ | \ | \ | 1.5^E+6^ ± 4.4^E+5^ |
| **MSH1_10^7^+K169_10^5^** | 0.03 ± 0.002* | 5.96 ± 0.73 | 18.56 ± 0.80* | 8.1^E+7^ ± 2.0^E+7^ | 5.7^E+6^ ± 1.4^E+6^* |
| **K169_10^4^** | \ | \ | \ | \ | 1.7^E+6^ ± 3.8^E+5^ |
| **MSH1_10^7^+K169_10^4^** | 0.15 ± 0.054 | 4.09 ± 0.50* | 12.35 ± 0.87* | 8.8^E+7^ ± 2.8^E+7^ | 1.1^E+6^ ± 3.5^E+5^ |
| **K169_10^3^** | \ | \ | \ | \ | 1.4^E+5^ ± 7.6^E+4^ |
| **MSH1_10^7^+K169_10^3^** | 0.24 ± 0.028 | 4.54 ± 0.60* | 13.97 ± 0.92* | 8.0^E+7^ ± 1.0^E+7^ | 1.5^E+5^ ± 1.1^E+5^ |
| **MSH1_10^5^** | 0.20 ± 0.084 | 5.08 ± 0.41 | 14.48 ± 1.89 | 7.2^E+7^ ± 2.4^E+7^ | \ |
| **MSH1_10^5^+K169_10^7^** | 0.41 ± 0.075 | 7.38 ± 0.20* | 36.55 ± 2.68* | 5.6^E+7^ ± 2.6^E+7^ | 1.7^E+7^ ± 2.8^E+6^* |
| **MSH1_10^4^** | 0.14 ± 0.023 | 4.99 ± 0.32 | 14.97 ± 1.36 | 5.5^E+7^ ± 1.5^E+7^ | \ |
| **MSH1_10^4^+K169_10^7^** | 0.57 ± 0.068* | 6.71 ± 0.49* | 25.13 ± 1.82* | 1.2^E+7^ ± 8.6^E+5^* | 1.6^E+7^ ± 2.9^E+6^* |
| **MSH1_10^3^** | 0.27 ± 0.060 | 4.37 ± 0.23 | 19.61 ± 1.14 | 1.4^E+7^ ± 2.3^E+6^ | \ |
| **MSH1_10^3^+K169_10^7^** | 0.92 ± 0.143* | 7.94 ± 0.58* | 35.90 ± 4.66* | 1.5^E+7^ ± 5.2^E+6^ | 1.5^E+7^ ± 1.8^E+6^* |

Table S2.2. Calculated fold changes of the BAM mineralization/cell density parameters between dual-species systems and corresponding mono-species systems for different MSH1/K169 initial cell densities. The black asterisk indicates parameters that significantly differ (p value < 0.05) between dual-species (*R_T_* = 2) and mono-species (*R_T_* = 1) systems for a certain initial cell density.

|  | ***λ* (h)** | ***µ* (%/h)** | ***A* (%)** | ***D_MSH1_* (CFU/mL)** | ***D_K169_* (CFU/mL)** |
| --- | --- | --- | --- | --- | --- |
| **MSH1_10^7^+K169_10^7^** | 0.06* | 1.08* | 0.70* | 1.10 | 5.74* |
| **MSH1_10^7^+K169_10^5^** | 0.09* | 0.86 | 0.81* | 1.11 | 3.70* |
| **MSH1_10^7^+K169_10^4^** | 0.55 | 0.59* | 0.54* | 1.21 | 0.67 |
| **MSH1_10^7^+K169_10^3^** | 0.85 | 0.66* | 0.61* | 1.10 | 1.03 |
| **MSH1_10^5^+K169_10^7^** | 2.04 | 1.45* | 2.52* | 0.78 | 8.99* |
| **MSH1_10^4^+K169_10^7^** | 3.97* | 1.35* | 1.68* | 0.22* | 8.51* |
| **MSH1_10^3^+K169_10^7^** | 3.43* | 1.82* | 1.83* | 1.05 | 8.14* |

| 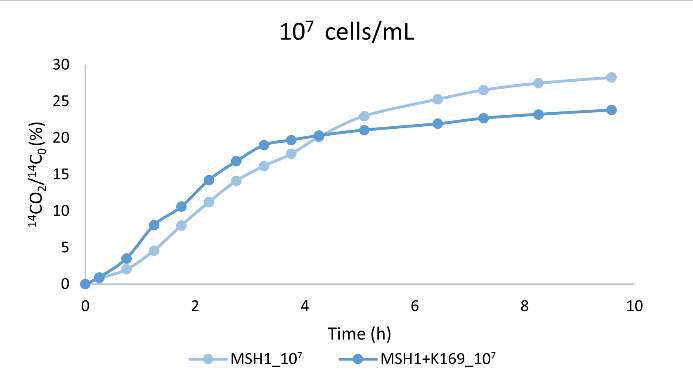  a | 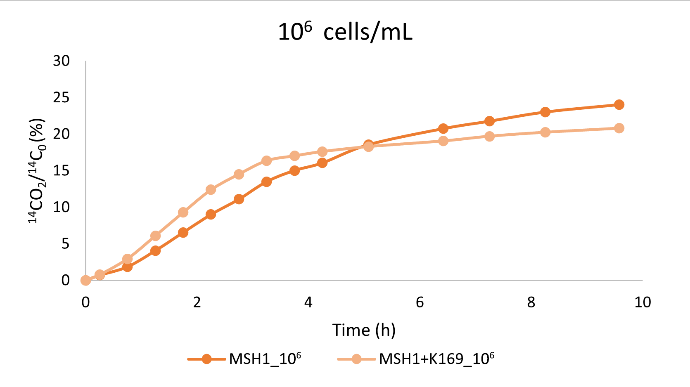  b |
| --- | --- |
| 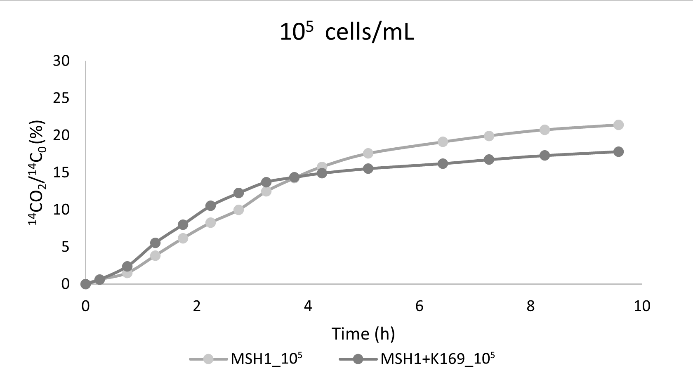  c | 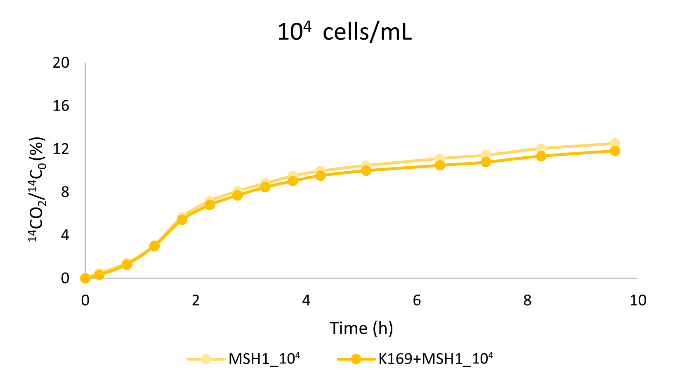  d |

Figure S2.3. Cumulative BAM mineralization curves at day 7 (measured cumulative ^14^CO_2_ production relative to the initial amount of ^14^C-BAM added (^14^C_0_)) obtained for dual-species systems of *A. niigataensis* MSH1 and *Piscinibacter* sp. K169 (*R_T_* = 2) and for MSH1 in mono-species systems (*R_T_* = 1) at identical starting cell density for MSH1 and K169 of 10^7^ cells/mL (panel a), 10^6^ cells/mL (panel b), 10^5^ cells/mL (panel c), and 10^4^ cells/mL (panel d). The different colors refer to the strain combinations as indicated below the graph. The numbers after the strain names refer to the initial cell densities at day 0. Values are averages of four replicates. Standard deviations are not shown for clarity but never exceeded 0.4%. The color of each curve corresponds to the bar under the same condition in main text Figure 2.

Table S2.3. Values of the BAM mineralization parameters and cell densities of MSH1/K169 obtained for dual-species systems of MSH1 and K169 (*R_T_* = 2) at identical initial cell densities of both MSH1 and K169 of 10^7^ to 10^4^ cells/mL and for MSH1/K169 mono-species systems (*R_T_* = 1) at initial cell densities of 10^7^ cells/mL to 10^4^ cells/mL. The numbers after the strain names refer to the nominal initial cell densities at day 0. The green numbers indicate a significant positive effect of K169 on the BAM mineralization/cell density parameter. The black asterisk indicates values that significantly differ between dual-species systems (*R_T_* = 2) and MSH1/K169 mono-species systems (*R_T_* = 1) with the same initial MSH1 cell density (p value < 0.05). The values are average values ± standard deviation of four replicates.

|  | *λ* (h) | *µ* (%/h) | *A* (%) | *D_MSH1_* (CFU/mL) | *D_K169_* (CFU/mL) |
| --- | --- | --- | --- | --- | --- |

| **10^7^** |  |  |  |  |  |
| --- | --- | --- | --- | --- | --- |
| **MSH1** | 0.46 ± 0.04 | 5.83 ± 0.24 | 28.17 ± 1.74 | 3.3^E+8^ ± 7.4^E+7^ | \ |
| **K169** | \ | \ | \ | \ | 3.4^E+7^ ± 5.2^E+6^ |
| **MSH1+K169** | 0.26 ± 0.03* | 7.21 ± 0.26* | 22.79 ± 1.26* | 3.4^E+8^ ± 7.1^E+7^ | 7.2^E+7^ ± 1.5^E+7^* |
| **10^6^** |  |  |  |  |  |
| **MSH1** | 0.38 ± 0.04 | 4.51 ± 0.32 | 24.09 ± 1.35 | 3.2^E+8^ ± 6.8^E+7^ | \ |
| **K169** | \ | \ | \ | \ | 3.7^E+7^ ± 5.5^E+6^ |
| **MSH1+K169** | 0.20 ± 0.03* | 6.28 ± 0.40* | 19.87 ± 1.46* | 3.8^E+8^ ± 5.4^E+7^ | 3.3^E+8^ ± 7.4^E+7^* |
| **10^5^** |  |  |  |  |  |
| **MSH1** | 0.48 ± 0.04 | 4.51 ± 0.23 | 21.30 ± 1.12 | 3.3^E+8^ ± 7.0^E+7^ | \ |
| **K169** | \ | \ | \ | \ | 3.8^E+7^ ± 5.6^E+6^ |
| **MSH1+K169** | 0.26 ± 0.04* | 5.20 ± 0.19* | 16.95 ± 1.27* | 3.2^E+8^ ± 4.5^E+7^ | 7.2^E+7^ ± 1.5^E+7^* |
| **10^4^** |  |  |  |  |  |
| **MSH1** | 0.21 ± 0.02 | 3.21 ± 0.27 | 11.90 ± 1.40 | 3.1^E+8^ ± 6.2^E+7^ | \ |
| **K169** | \ | \ | \ | \ | 1.0^E+7^ ± 0.0^E+0^ |
| **MSH1+K169** | 0.26 ± 0.03 | 3.17 ± 0.47 | 11.14 ± 1.35 | 3.4^E+8^ ± 3.7^E+7^ | 3.7^E+6^ ± 2.2^E+6^* |

Table S2.4. Calculated fold changes of the BAM mineralization/cell density parameters between dual-species systems and corresponding mono-species systems in systems in which K169 and MSH1 are inoculated at identical initial cell densities (from 10^7^ cells/mL to 10^4^ cells/mL). The black asterisk indicates values that significantly differ between dual-species systems (*R_T_* = 2) and MSH1/K169 mono-species systems (*R_T_* = 1) with the same initial MSH1 cell density (p value < 0.05).

|  | ***λ* (h)** | ***µ* (%/h)** | ***A* (%)** | ***D_MSH1_* (CFU/mL)** | ***D_K169_* (CFU/mL)** |
| --- | --- | --- | --- | --- | --- |
| **MSH1 10^7^ + K169_10^7^** | 0.55* | 1.24* | 0.81* | 1.03 | 2.10* |

| **MSH1 10^6^ + K169_10^6^** | 0.53* | 1.39* | 0.82* | 1.19 | 1.91* |
| --- | --- | --- | --- | --- | --- |
| **MSH1 10^5^ + K169_10^5^** | 0.54* | 1.15* | 0.80* | 0.97 | 1.92* |
| **MSH1 10^4^ + K169_10^4^** | 1.19 | 0.99 | 0.94 | 1.10 | 0.37* |

**Document S3. Impact** **of acetate concentration**


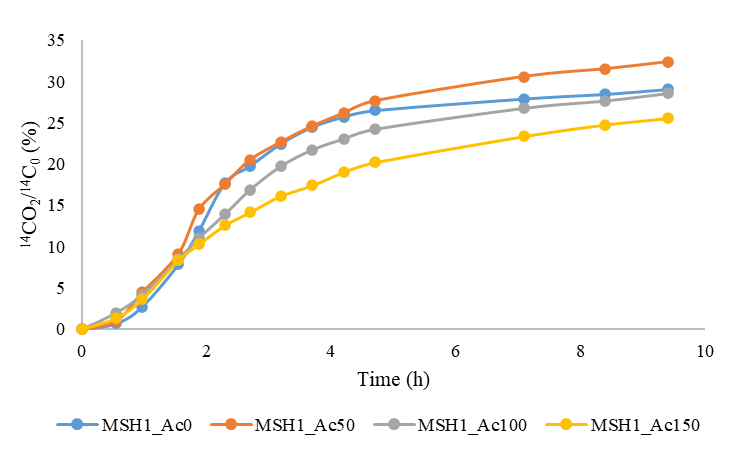


Figure S3.1. Cumulative BAM mineralization curves at day 7 (measured cumulative ^14^CO_2_ production relative to the initial amount of ^14^C-BAM added (^14^C_0_)) obtained for *A. niigataensis* MSH1 mono-species (*R_T_* = 1) in sand microcosms supplemented with MMO with 0 µg/L to 150 µg/L Ac. The different colors refer to the strain combinations as indicated below the graph. Values are averages of four replicates. Standard deviations are not shown for clarity but never reached more than 0.4%. The color of each curve corresponds to the bar under the same condition in main text Figure 3.

Table S3.1. Values of the BAM mineralization parameters and cell densities of MSH1/K169 obtained for dual-species systems of MSH1 and K169 (*R_T_* = 2) and for MSH1/K169 in mono-species systems (*R_T_* = 1) in sand microcosms supplemented with MMO with 150 µg/L Ac, MMO with 100 µg/L Ac, MMO with 50 µg/L Ac, and MMO with 0 µg/L Ac. The green numbers indicate a significant positive effect of K169 on the BAM mineralization parameter within each condition. The black asterisk indicates values that significantly differ between dual-species (*R_T_* = 2) and MSH1/K169 mono-species (*R_T_* = 1) systems (p value < 0.05) amended with the same concentration of Ac. Values are average values ± standard deviation of four replicates.

|  | ***λ* (h)** | ***µ* (%/h)** | ***A* (%)** | ***D_MSH1_* (CFU/mL)** | ***D_K169_* (CFU/mL)** |
| --- | --- | --- | --- | --- | --- |
| **150 µg/L Ac** |  |  |  |  |  |
| **MSH1** | 0.43 ± 0.02 | 7.01 ± 0.28 | 25.57 ± 0.36 | 7.3^E+7^ ± 2.8^E+7^ | \ |

| **K169** | \ | \ | \ | \ | 1.2^E+5^ ± 0.0^E+0^ |
| --- | --- | --- | --- | --- | --- |
| **MSH1+K169** | 0.05 ± 0.003* | 8.24 ± 0.20* | 24.78 ± 0.16* | 9.7^E+7^ ± 4.6^E+6^ | 2.6^E+6^ ± 9.5^E+5^* |
| **100 µg/L Ac** |  |  |  |  |  |
| **MSH1** | 0.50 ± 0.02 | 8.05 ± 0.39 | 27.07 ± 0.62 | 7.7^E+7^ ± 1.4^E+7^ | \ |
| **K169** | \ | \ | \ | \ | 9.3^E+3^ ± 2.3^E+3^ |
| **MSH1+K169** | 0.27 ± 0.03* | 10.48 ± 0.44* | 27.90 ± 0.79 | 1.0^E+8^ ± 1.4^E+7^ | 7.0^E+6^ ± 2.0^E+6^* |
| **50 µg/L Ac** |  |  |  |  |  |
| **MSH1** | 0.64 ± 0.04 | 11.01 ± 0.82 | 31.88 ± 1.39 | 8.3^E+7^ ± 1.6^E+7^ | \ |
| **K169** | \ | \ | \ | \ | 1.2^E+4^ ± 1.7^E+4^ |
| **MSH1+K169** | 0.50 ± 0.03* | 13.53 ± 0.43* | 28.66 ± 0.62* | 7.5^E+7^ ± 8.2^E+6^ | 1.5^E+6^ ± 6.8^E+5^* |
| **0 µg/L Ac** |  |  |  |  |  |
| **MSH1** | 0.87 ± 0.06 | 12.17 ± 0.57 | 28.53 ± 0.93 | 8.5^E+7^ ± 1.9^E+7^ | \ |
| **K169** | \ | \ | \ | \ | 2.4^E+4^ ± 5.7^E+3^ |
| **MSH1+K169** | 0.52 ± 0.05* | 12.01 ± 0.81 | 26.81 ± 0.55* | 7.0^E+7^ ± 2.7^E+7^ | 2.0^E+6^ ± 5.7^E+5^* |

Table S3.2. Calculated fold changes of the BAM mineralization/cell density parameters between dual-species and respective mono-species systems in sand microcosms with MMO supplemented with Ac at concentration from 150 µg/L to 0 µg/L. The black asterisk indicates values that significantly differ between dual-species (*R_T_* = 2) and MSH1/K169 mono-species (*R_T_* = 1) systems (p value < 0.05) amended with the same Ac concentration.

|  | ***λ* (h)** | ***µ* (%/h)** | ***A* (%)** | ***D_MSH1_* (CFU/mL)** | ***D_K169_* (CFU/mL)** |
| --- | --- | --- | --- | --- | --- |
| **150 µg/L Ac** | 0.12* | 1.18* | 0.97* | 1.33 | 21.67* |
| **100 µg/L Ac** | 0.54* | 1.30* | 1.03 | 1.32 | 750.00* |
| **50 µg/L Ac** | 0.78* | 0.78* | 1.23* | 0.90 | 70.31* |
| **0 µg/L Ac** | 0.59* | 0.59 | 0.99* | 0.82 | 83.33* |

**Document S4. Impact of sand organic carbon**

| 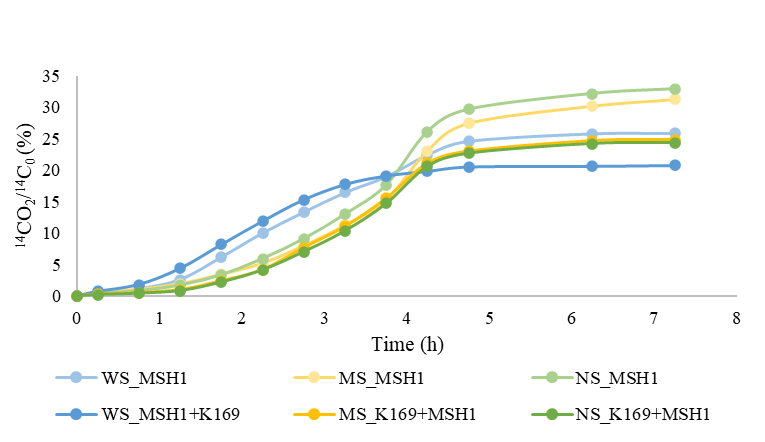  a | 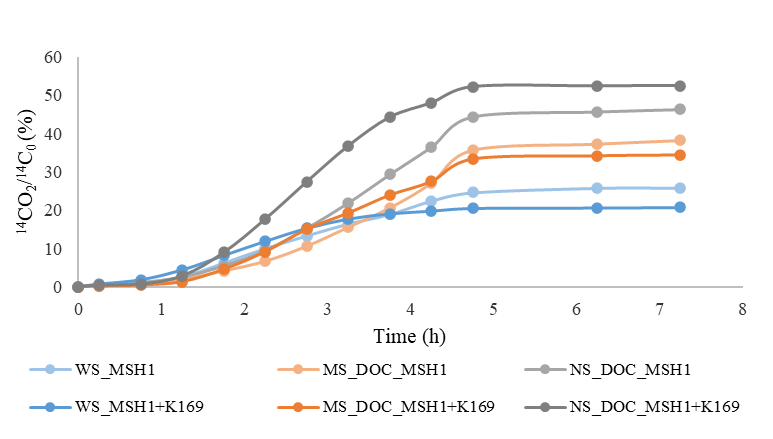  b |
| --- | --- |

Figure S4.1. Cumulative BAM mineralization curves at day 7 (measured cumulative ^14^CO_2_ production relative to the initial amount of ^14^C-BAM added (^14^C_0_)) obtained for dual-species systems of *A*. *niigataensis* MSH1 and *Piscinibacter* sp. K169 (*R_T_* = 2) and for MSH1 in mono-species systems (*R_T_* = 1) in the experiment that assesses the role of sand organic carbon and the sand matrix on the mutualistic interactions between MSH1 and K169. Panel a: systems with normal sand (WS), muffled sand (MS) and no sand (NS); Panel b: systems with muffled sand (MS) or no sand (NS) and added DOC extract (DOC). The different colors refer to the strain combinations and experimental conditions as indicated below the graph. Values are average values of four replicates. Standard deviations are not shown for clarity but never exceeded 0.5%. The color of each curve corresponds to the bar under the same condition in the main text Figure 4.

Table S4.1. Values of the BAM mineralization parameters and cell densities of MSH1/K169 obtained for dual-species systems of MSH1 and K169 (*R_T_* = 2) and for MSH1/K169 mono-species systems (*R_T_* = 1) in the experiment that assess the role of sand organic carbon on the mutualistic interaction between MSH1 and K169. The abbreviations “WS”, “MS” and “NS” stand for the different tested conditions, i.e., “with sand”, “muffled sand” and “no sand” respectively. The green number indicates a significant positive effect of K169 on the BAM mineralization/cell density parameter within an experimental condition. The black asterisk indicates values that significantly differ between dual-species (*R_T_* = 2) and MSH1/K169 mono-species (*R_T_* = 1) systems (p value < 0.05) within each condition. Values are average values ± standard deviation of four replicates.

| **Conditions** | ***λ* (h)** | ***µ* (%/h)** | ***A* (%)** | ***D_MSH1_* (CFU/mL)** | ***D_K169_* (CFU/mL)** |
| --- | --- | --- | --- | --- | --- |
| **WS** |  |  |  |  |  |
| **MSH1** | 1.03 ± 0.07 | 7.96 ± 0.20 | 26.55 ± 0.80 | 7.1^E+6^ ± 2.4^E+6^ | \ |
| **K169** | \ | \ | \ | \ | 1.8^E+5^ ± 2.6^E+4^ |
| **K169+MSH1** | 0.71 ± 0.02* | 8.01 ±0.24 | 21.31 ± 1.26* | 3.2^E+6^ ± 1.9^E+6^ | 1.2^E+6^ ± 1.4^E+5^* |
| **MS_DOC** |  |  |  |  |  |
| **MSH1** | 1.82 ± 0.06 | 11.95 ± 0.51 | 40.30 ± 0.88 | 9.4^E+5^ ± 1.4^E+5^ | \ |
| **K169** | \ | \ | \ | \ | 1.9^E+5^ ± 2.8^E+4^ |
| **K169+MSH1** | 1.48 ± 0.09* | 11.58 ± 0.36 | 35.37 ± 2.07* | 4.0^E+5^ ± 0.0^E+0^* | 4.5^E+4^ ± 2.7^E+4^* |
| **NS_DOC** |  |  |  |  |  |
| **MSH1** | 1.71 ± 0.08 | 15.65 ± 0.67 | 47.99 ± 1.54 | 1.0^E+6^ ± 1.6^E+5^ | \ |
| **K169** | \ | \ | \ | \ | 1.5^E+5^ ± 2.3^E+4^ |
| **K169+MSH1** | 1.42 ± 0.07* | 21.85 ± 0.80* | 53.41 ± 1.08* | 4.4^E+5^ ± 0.0^E+0^* | 3.7^E+4^ ± 2.2^E+4^* |
| **MS** |  |  |  |  |  |
| **MSH1** | 1.87 ± 0.07 | 9.37 ± 0.38 | 33.35 ± 0.99 | 5.9^E+5^ ± 7.0^E+4^ | \ |
| **K169** | \ | \ | \ | \ | 1.5^E+5^ ± 2.2^E+4^ |
| **K169+MSH1** | 1.89 ± 0.10 | 9.15 ± 0.51 | 25.80 ± 0.89* | 7.1^E+4^ ± 1.7^E+4^* | 1.2^E+5^ ± 1.8^E+4^ |
| **NS** |  |  |  |  |  |
| **MSH1** | 1.86 ± 0.07 | 10.65 ± 0.38 | 34.58 ± 2.87 | 1.0^E+6^ ± 3.8^E+4^ | \ |
| **K169** | \ | \ | \ | \ | 1.8^E+4^ ± 2.6^E+3^ |
| **K169+MSH1** | 1.95 ± 0.08 | 9.08 ± 0.27 | 25.24 ± 0.92* | 6.4^E+4^ ± 1.6^E+4^* | 1.8^E+5^ ± 0.0^E+0^* |

Table S4.2. Calculated fold changes of the BAM mineralization/cell density parameters between dual-species and respective mono-species systems in the experiment that assesses the role of sand organic carbon on the mutualistic interaction between MSH1 and K169. The abbreviations “WS”, “MS” and “NS” stand for microcosms “with sand”, “muffled sand” and “no sand”, respectively. The black asterisk indicates values that significantly differ between dual-species (*R_T_* = 2) and MSH1/K169 mono-species (*R_T_* = 1) systems (p value < 0.05) within each condition.

|  | ***λ* (h)** | ***µ* (%/h)** | ***A* (%)** | ***D_MSH1_* (CFU/mL)** | ***D_K169_* (CFU/mL)** |
| --- | --- | --- | --- | --- | --- |
| **WS** | 0.69* | 1.01 | 0.80* | 0.45 | 6.50* |

| **MS_DOC** | 0.81* | 0.97 | 0.88* | 0.42* | 0.23* |
| --- | --- | --- | --- | --- | --- |
| **NS_DOC** | 0.83* | 1.40* | 1.11* | 0.42* | 0.24* |
| **MS** | 1.01 | 0.98 | 0.77* | 0.12* | 0.79 |
| **NS** | 1.05 | 0.85 | 0.73* | 0.06* | 10.14* |


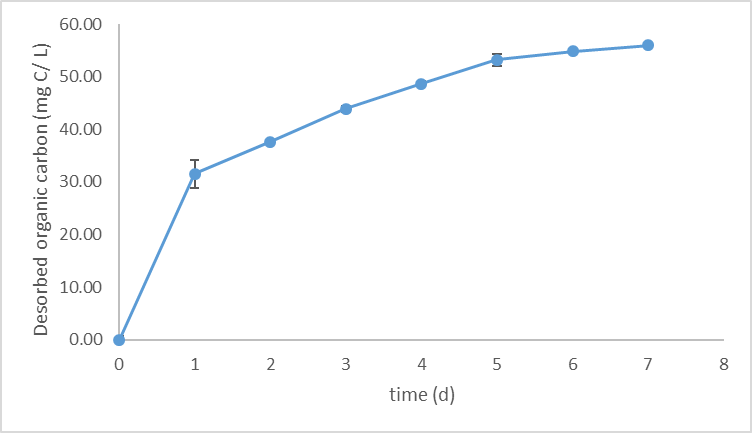


Figure S4.2. Cumulative desorption curve for organic carbon from the used sand. Mean values and standard deviations are based on three replicates.

**Document S5. Hypothetical scenarios illustrating the effect of initial K169/MSH1 cell densities and ratios on their cooperation in sand filter microcosms**

**
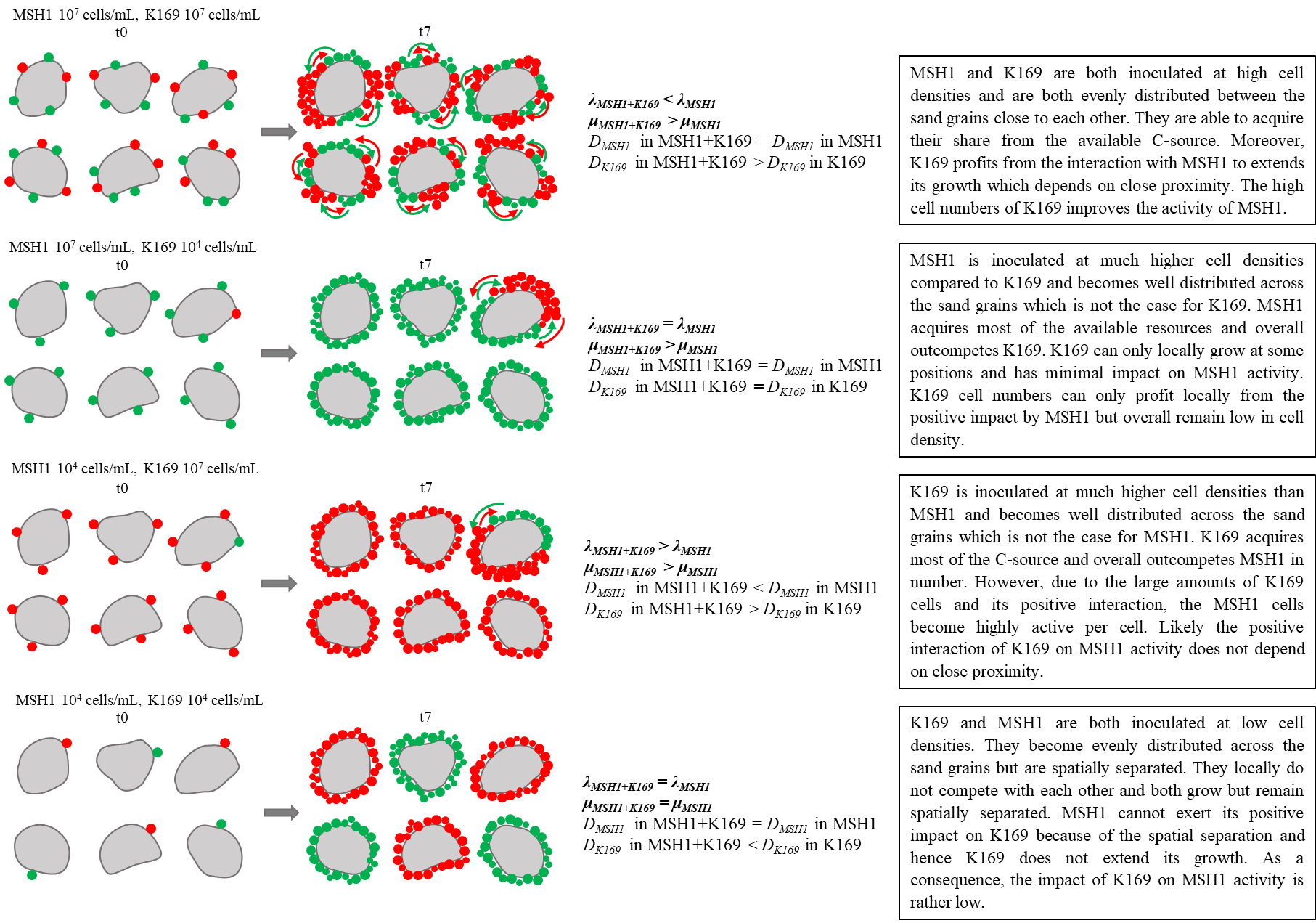
**

Figure S5. Illustration of hypothetical scenarios showing the effect of different initial K169/MSH1 cell density ratios and cell densities on K169/MSH1 cooperation in the sand filter microcosms. The left side of the figure marked as t_0_ illustrates the situation and cell distribution directly after inoculation of different cell densities of K169 and MSH1 and different MSH1/K169 cell density ratios. The right side shows the situation and cell distribution at t_7_ under the different initial cell density ratios. Grey irregular shapes represent sand grains; green dots represent MSH1 cells, and red dots represent K169 cells. Arrows indicate the cooperative interactions in both directions (red: K169 positively impacts BAM mineralization by MSH1; green: MSH1 positively impacts K169 growth).
